# Supplementary material for: "Long-term effects of center volume on transplant outcomes in adult kidney transplant recipients"
Source: PLoS One. 2024 Jun 6;19(6):e0301425. doi: 10.1371/journal.pone.0301425 (PMC11156332; doi:10.1371/journal.pone.0301425)
Supplement: S1 Appendix — (DOCX) [file pone.0301425.s001.docx]

**Supplementary Appendix:**

**Table S1.** Characteristics of De-novo Adult Kidney Transplant Recipients of KDPI>85% Deceased Donor Kidneys

|  | Overall  (n=10225) | Low  (Q1; n=2098) | Medium  (Q2; n=2851) | Medium-High  (Q3; n=3013) | High  (Q4; n=2263) | P-value ^*^ |
| --- | --- | --- | --- | --- | --- | --- |
| **Recipient Characteristics** |  |  |  |  |  |  |
| Recipient Age, Years | 60.48 ± 10.48 | 60.18 ± 10.70 | 60.87 ± 10.41 | 60.34 ± 10.44 | 60.45 ± 10.43 | 0.178 |
| Recipient Sex, Male | 6437 (62.95%) | 1325 (63.16%) | 1815 (63.66%) | 1866 (61.93%) | 1431 (63.23%) | 0.557 |
| AA Recipient Ethnicity | 3658 (35.93%) | 644 (30.89%) | 1168 (41.17%) | 1094 (36.43%) | 752 (33.35%) | <0.001 |
| Missing | 45 (0.4%) | 13 (0.6%) | 14 (0.5%) | 10 (0.3%) | 8 (0.4%) |  |
| Recipient BMI | 27.72 ± 5.08 | 27.79 ± 5.08 | 28.02 ± 5.09 | 27.49 ± 4.91 | 27.58 ± 5.29 | <0.001 |
| Missing | 73 (0.7%) | 10 (0.5%) | 16 (0.6%) | 14 (0.5%) | 33 (1.5%) |  |
| Recipient Diabetes, Y | 4533 (44.37%) | 949 (45.23%) | 1263 (44.35%) | 1356 (45.00%) | 965 (42.74%) | 0.312 |
| Missing | 8 (0.1%) | 0 (0%) | 3 (0.1%) | 0 (0%) | 5 (0.2%) |  |
| Recipient Peak PRA | 9.35 ± 22.00 | 9.16 ± 21.64 | 8.51 ± 20.91 | 10.58 ± 23.52 | 8.92 ± 21.51 | <0.001 |
| Missing | 253 (2.5%) | 26 (1.2%) | 83 (2.9%) | 58 (1.9%) | 86 (3.8%) |  |
| Recipient Kidney CIT | 19.60 ± 8.86 | 18.17 ± 7.59 | 17.98 ± 7.48 | 20.15 ± 9.26 | 22.09 ± 10.16 | <0.001 |
| Missing | 528 (5.2%) | 165 (7.9%) | 191 (6.7%) | 98 (3.3%) | 74 (3.3%) |  |
| Recipient Hepatitis C Antibody, Positive | 722 (7.57%) | 142 (7.08%) | 206 (7.84%) | 207 (7.26%) | 167 (8.15%) | 0.506 |
| Missing | 692 (6.8%) | 93 (4.4%) | 222 (7.8%) | 162 (5.4%) | 215 (9.5%) |  |
| Recipient Dialysis at TX, Y | 9239 (90.96%) | 1942 (93.14%) | 2591 (91.65%) | 2683 (89.43%) | 2023 (90.11%) | <0.001 |
| Missing | 68 (0.7%) | 13 (0.6%) | 24 (0.8%) | 13 (0.4%) | 18 (0.8%) |  |
| HLA antigen mismatch | 4.49 ± 1.34 | 4.41 ± 1.38 | 4.54 ± 1.32 | 4.47 ± 1.37 | 4.54 ± 1.28 | 0.006 |
| Missing | 3 (<0.1%) | 1 (<0.1%) | 2 (0.1%) | 0 (0%) | 0 (0%) |  |
| Re-transplant, Y | 573 (5.60%) | 117 (5.58%) | 176 (6.17%) | 131 (4.35%) | 149 (6.58%) | 0.002 |
| **Immunosuppression** |  |  |  |  |  |  |
| T-cell Depletion Induction | 5840 (57.11%) | 1150 (54.81%) | 1586 (55.63%) | 1801 (59.77%) | 1303 (57.58%) | 0.001 |
| IL-2RA Induction | 2136 (20.89%) | 543 (25.88%) | 500 (17.54%) | 687 (22.80%) | 406 (17.94%) | <0.001 |
| Calcineurin Inhibitor | 8891 (86.95%) | 1823 (86.89%) | 2489 (87.30%) | 2752 (91.34%) | 1827 (80.73%) | <0.001 |
| Maintenance Steroids | 7024 (68.69%) | 1556 (74.17%) | 2006 (70.36%) | 2220 (73.68%) | 1242 (54.88%) | <0.001 |
| **Donor Characteristics** |  |  |  |  |  |  |
| Donor Age, Years | 60.58 ± 12.13 | 61.92 ± 9.84 | 61.74 ± 9.70 | 58.80 ± 14.90 | 60.25 ± 12.37 | <0.001 |
| Donor Sex, Male | 4391 (42.94%) | 865 (41.23%) | 1235 (43.32%) | 1316 (43.68%) | 975 (43.08%) | 0.34 |
| AA Donor Ethnicity | 3074 (30.30%) | 515 (24.69%) | 919 (32.58%) | 981 (32.78%) | 659 (29.37%) | <0.001 |
| Missing | 81 (0.8%) | 12 (0.6%) | 30 (1.1%) | 20 (0.7%) | 19 (0.8%) |  |
| Donor BMI | 28.10 ± 7.05 | 28.02 ± 6.61 | 28.25 ± 6.91 | 28.16 ± 7.50 | 27.90 ± 7.01 | 0.437 |
| Missing | 14 (0.1%) | 5 (0.2%) | 3 (0.1%) | 2 (0.1%) | 4 (0.2%) |  |
| Donor Diabetes, Y | 2704 (26.50%) | 506 (24.13%) | 750 (26.35%) | 858 (28.57%) | 590 (26.13%) | 0.005 |
| Missing | 21 (0.2%) | 1 (<0.1%) | 5 (0.2%) | 10 (0.3%) | 5 (0.2%) |  |
| Donor Hypertension, Y | 7884 (77.26%) | 1625 (77.53%) | 2232 (78.45%) | 2289 (76.20%) | 1738 (76.90%) | 0.214 |
| Missing | 20 (0.2%) | 2 (0.1%) | 6 (0.2%) | 9 (0.3%) | 3 (0.1%) |  |
| Donor Hep C Antibody, Positive | 499 (4.88%) | 90 (4.29%) | 162 (5.68%) | 124 (4.12%) | 123 (5.44%) | 0.013 |
| Missing | 3 (<0.1%) | 2 (0.1%) | 1 (<0.1%) | 0 (0%) | 0 (0%) |  |
| Donor Don. After Cardiac Death, Y | 818 (8.00%) | 192 (9.15%) | 274 (9.61%) | 206 (6.84%) | 146 (6.45%) | <0.001 |
| Donor Terminal Creatinine | 1.24 ± 1.04 | 1.20 ± 0.99 | 1.23 ± 1.10 | 1.28 ± 1.01 | 1.25 ± 1.07 | <0.001 |
| **Outcomes** |  |  |  |  |  |  |
| Graft failure (5 yr) | 2404 (23.51%) | 474 (22.59%) | 719 (25.22%) | 690 (22.90%) | 521 (23.02%) | 0.088 |
| Death (5 yr) | 1766 (17.27%) | 400 (19.07%) | 526 (18.45%) | 463 (15.37%) | 377 (16.66%) | 0.001 |
| Recipient LOS, Days | 8.50 ± 12.69 | 9.13 ± 9.54 | 8.66 ± 9.53 | 6.97 ± 6.68 | 9.72 ± 21.55 | <0.001 |
| Missing | 38 (0.4%) | 9 (0.4%) | 7 (0.2%) | 12 (0.4%) | 10 (0.4%) |  |
| Follow-up (days) | 1851.08 ± 1395.46 | 1799.94 ± 1398.86 | 1815.10 ± 1376.36 | 1892.05 ± 1363.25 | 1889.28 ± 1455.48 | 0.019 |
| Treated for rejection within 1 year | 917 (12.79%) | 211 (14.09%) | 250 (12.74%) | 255 (11.47%) | 201 (13.51%) | 0.093 |
| Missing | 3054 (29.9%) | 600 (28.6%) | 889 (31.2%) | 790 (26.2%) | 775 (34.2%) |  |

Notes: Continuous variables summarized as mean±standard deviation; categorical variables, as count (percentage). “*” Testing the difference between all four groups.

Abbreviations: AA, African American; BMI, Body mass index; CIT, Cold ischemia time; LOS, Length of stay; TX, Transplant; IL-2RA, Interleukin-2 receptor antagonist; HLA, Human leukocyte antigen; PRA, Panel reactive antibody; Y, Yes

**Table S2.** Characteristics of Adult Re-Transplant Kidney Recipients of Deceased Donor Kidneys

|  | Overall  (n=17760) | Low  (Q1; n=4260) | Medium  (Q2; 4786) | Medium-High  (Q3; n=4678) | High  (Q4; n=4036) | P-value ^*^ |
| --- | --- | --- | --- | --- | --- | --- |
| **Recipient Characteristics** |  |  |  |  |  |  |
| Recipient Age, Years | 45.73 ± 12.66 | 45.17 ± 12.65 | 45.90 ± 12.60 | 45.68 ± 12.65 | 46.21 ± 12.73 | 0.0050 |
| Recipient Sex, Male | 10512 (59.19%) | 2551 (59.88%) | 2857 (59.69%) | 2722 (58.19%) | 2382 (59.02%) | 0.3410 |
| AA Recipient Ethnicity | 4747 (26.88%) | 1019 (24.08%) | 1384 (29.03%) | 1353 (29.10%) | 991 (24.70%) | <0.001 |
| Missing | 99 (0.6%) | 28 (0.7%) | 19 (0.4%) | 28 (0.6%) | 24 (0.6%) |  |
| Recipient BMI | 26.45 ± 5.32 | 26.61 ± 5.30 | 26.56 ± 5.44 | 26.25 ± 5.21 | 26.40 ± 5.29 | 0.006 |
| Missing | 186 (1.0%) | 18 (0.4%) | 26 (0.5%) | 35 (0.7%) | 107 (2.7%) |  |
| Recipient Diabetes, Y | 3403 (19.30%) | 824 (19.49%) | 875 (18.42%) | 909 (19.52%) | 795 (19.87%) | 0.326 |
| Missing | 124 (0.7%) | 32 (0.8%) | 35 (0.7%) | 22 (0.5%) | 35 (0.9%) |  |
| Recipient Peak PRA | 56.72 ± 39.95 | 55.31 ± 40.32 | 55.77 ± 40.18 | 60.72 ± 39.24 | 54.72 ± 39.80 | <0.001 |
| Missing | 308 (1.7%) | 39 (0.9%) | 70 (1.5%) | 107 (2.3%) | 92 (2.3%) |  |
| Recipient Kidney CIT | 18.08 ± 8.14 | 17.20 ± 7.44 | 17.26 ± 7.50 | 18.24 ± 8.11 | 19.73 ± 9.23 | <0.001 |
| Missing | 1043 (5.9%) | 306 (7.2%) | 341 (7.1%) | 254 (5.4%) | 142 (3.5%) |  |
| Recipient Hepatitis C Antibody, Positive | 1181 (7.20%) | 283 (7.01%) | 314 (7.14%) | 310 (7.01%) | 274 (7.74%) | 0.5720 |
| Missing | 1360 (7.7%) | 222 (5.2%) | 386 (8.1%) | 258 (5.5%) | 494 (12.2%) |  |
| Recipient Dialysis at TX, Y | 15846 (89.99%) | 3904 (92.23%) | 4242 (89.89%) | 4159 (89.38%) | 3541 (88.46%) | <0.001 |
| Missing | 152 (0.9%) | 27 (0.6%) | 67 (1.4%) | 25 (0.5%) | 33 (0.8%) |  |
| HLA antigen mismatch | 3.26 ± 1.94 | 3.21 ± 1.95 | 3.33 ± 1.92 | 3.19 ± 1.93 | 3.31 ± 1.94 | <0.001 |
| Missing | 9 (0.1%) | 1 (<0.1%) | 7 (0.1%) | 0 (0%) | 1 (<0.1%) |  |
| **Immunosuppression** |  |  |  |  |  |  |
| T-cell Depletion Induction | 12263 (69.05%) | 2770 (65.02%) | 3061 (63.96%) | 3497 (74.75%) | 2935 (72.72%) | <0.001 |
| IL-2RA Induction | 2211 (12.45%) | 734 (17.23%) | 553 (11.55%) | 477 (10.20%) | 447 (11.08%) | <0.001 |
| Calcineurin Inhibitor | 15668 (88.22%) | 3805 (89.32%) | 4196 (87.67%) | 4246 (90.77%) | 3421 (84.76%) | <0.001 |
| Maintenance Steroids | 14230 (80.12%) | 3501 (82.18%) | 3948 (82.49%) | 3969 (84.84%) | 2812 (69.67%) | <0.001 |
| **Donor Characteristics** |  |  |  |  |  |  |
| Donor Age, Years | 35.59 ± 15.21 | 35.11 ± 14.98 | 35.60 ± 15.40 | 35.17 ± 15.10 | 36.57 ± 15.33 | <0.001 |
| Donor Sex, Male | 10832 (60.99%) | 2584 (60.66%) | 2914 (60.89%) | 2912 (62.25%) | 2422 (60.01%) | 0.1740 |
| AA Donor Ethnicity | 2236 (12.67%) | 452 (10.64%) | 635 (13.40%) | 649 (13.94%) | 500 (12.48%) | <0.001 |
| Missing | 110 (0.6%) | 11 (0.3%) | 47 (1.0%) | 24 (0.5%) | 28 (0.7%) |  |
| Donor BMI | 26.78 ± 6.62 | 26.43 ± 6.30 | 26.78 ± 6.48 | 26.84 ± 6.69 | 27.09 ± 7.02 | 0.006 |
| Missing | 31 (0.2%) | 9 (0.2%) | 8 (0.2%) | 8 (0.2%) | 6 (0.1%) |  |
| Donor Diabetes, Y | 933 (5.28%) | 179 (4.22%) | 252 (5.28%) | 257 (5.53%) | 245 (6.10%) | 0.001 |
| Missing | 77 (0.4%) | 15 (0.4%) | 13 (0.3%) | 29 (0.6%) | 20 (0.5%) |  |
| Donor Hypertension, Y | 3567 (20.21%) | 771 (18.20%) | 959 (20.13%) | 958 (20.65%) | 879 (21.92%) | <0.001 |
| Missing | 112 (0.6%) | 24 (0.6%) | 23 (0.5%) | 39 (0.8%) | 26 (0.6%) |  |
| Donor Hep C Antibody, Positive | 349 (1.97%) | 57 (1.34%) | 105 (2.20%) | 65 (1.39%) | 122 (3.03%) | <0.001 |
| Missing | 22 (0.1%) | 8 (0.2%) | 3 (0.1%) | 7 (0.1%) | 4 (0.1%) |  |
| Donor Don. After Cardiac Death, Y | 1930 (10.87%) | 439 (10.31%) | 568 (11.87%) | 470 (10.05%) | 453 (11.22%) | 0.018 |
| Missing | 1 (<0.1%) | 0 (0%) | 0 (0%) | 0 (0%) | 1 (<0.1%) |  |
| Donor Terminal Creatinine | 1.09 ± 0.86 | 1.03 ± 0.82 | 1.07 ± 0.87 | 1.14 ± 0.93 | 1.11 ± 0.80 | <0.001 |
| **Outcomes** |  |  |  |  |  |  |
| Graft failure (5 yr) | 3017 (16.99%) | 691 (16.22%) | 845 (17.66%) | 751 (16.05%) | 730 (18.09%) | 0.022 |
| Death (5 yr) | 1586 (8.93%) | 385 (9.04%) | 434 (9.07%) | 392 (8.38%) | 375 (9.29%) | 0.464 |
| Follow-up (days) | 2286.41 ± 1545.75 | 2294.49 ± 1613.78 | 2298.56 ± 1535.23 | 2289.75 ± 1476.34 | 2259.61 ± 1563.57 | 0.2710 |
| Recipient LOS, Days | 8.09 ± 11.26 | 8.83 ± 11.88 | 7.91 ± 9.95 | 7.08 ± 9.17 | 8.69 ± 13.86 | <0.001 |
| Missing | 61 (0.3%) | 17 (0.4%) | 11 (0.2%) | 15 (0.3%) | 18 (0.4%) |  |
| Treated for rejection within 1 year | 1793 (13.70%) | 434 (13.48%) | 446 (12.82%) | 457 (12.99%) | 456 (15.90%) | 0.0010 |
| Missing | 4674 (26.3%) | 1040 (24.4%) | 1307 (27.3%) | 1159 (24.8%) | 1168 (28.9%) |  |

Notes: Continuous variables summarized as mean±standard deviation; categorical variables, as count (percentage). “*” Testing the difference between all four groups.

“**” reported only for deceased donor kidneys.

Abbreviations: AA, African American; BMI, Body mass index; CIT, Cold ischemia time; LOS, Length of stay; TX, Transplant; IL-2RA, Interleukin-2 receptor antagonist; HLA, Human leukocyte antigen; PRA, Panel reactive antibody; Y, Yes

**Table S3.** Competing risk regression models for graft failure and patient death controlling all other covariates (deceased donors).

|  | Graft failure (frailty p-val < 0.001) | | Patient death (frailty p-val < 0.001) | |
| --- | --- | --- | --- | --- |
|  | HR (95% CI) | P-value | HR (95% CI) | P-value |
| Volume groups (ref = “Low”) |  |  |  |  |
| Medium | 1.007 (0.920, 1.103) | 0.873 | 0.954 (0.875, 1.041) | 0.292 |
| Medium-High | 0.922 (0.832, 1.021) | 0.118 | 0.856 (0.778, 0.943) | 0.002 |
| High | 0.993 (0.877, 1.125) | 0.915 | 0.932 (0.831, 1.045) | 0.229 |
| Recipient Age, Years | 0.975 (0.973, 0.977) | <0.001 | 1.053 (1.050, 1.055) | <0.001 |
| Recipient Sex, Male | 1.067 (1.021, 1.114) | 0.004 | 1.194 (1.135, 1.255) | <0.001 |
| Recipient Ethnicity (ref = “Caucasian”) |  |  |  |  |
| AA | 1.452 (1.38, 1.528) | <0.001 | 0.792 (0.747, 0.839) | <0.001 |
| Others | 0.904 (0.847, 0.966) | 0.003 | 0.616 (0.574, 0.661) | <0.001 |
| Recipient BMI | 1.013 (1.009, 1.017) | <0.001 | 0.992 (0.987, 0.996) | 0.001 |
| Recipient Diabetes, Y | 1.106 (1.055, 1.16) | <0.001 | 1.791 (1.707, 1.879) | <0.001 |
| Recipient Peak PRA | 1.002 (1.002, 1.003) | <0.001 | 1.001 (1.000, 1.002) | 0.052 |
| Recipient Kidney CIT | 1.004 (1.001, 1.006) | 0.005 | 1.004 (1.001, 1.007) | 0.006 |
| Recipient Hepatitis C Antibody, Positive | 1.234 (1.123, 1.357) | <0.001 | 1.385 (1.247, 1.538) | <0.001 |
| Recipient Dialysis at TX, Y | 1.437 (1.316, 1.569) | <0.001 | 1.514 (1.387, 1.652) | <0.001 |
| Recipient LOS, Days | 1.003 (1.002, 1.004) | <0.001 | 1.005 (1.004, 1.006) | <0.001 |
| Treated for rejection within 1 year (kidney) | 2.718 (2.586, 2.856) | <0.001 | 1.357 (1.261, 1.461) | <0.001 |
| Donor Age, Years | 1.016 (1.014, 1.017) | <0.001 | 1.005 (1.004, 1.007) | <0.001 |
| Donor Sex, Male | 0.983 (0.937, 1.031) | 0.471 | 1.035 (0.981, 1.092) | 0.204 |
| Donor Ethnicity (ref = “Caucasian”) |  |  |  |  |
| AA | 1.348 (1.274, 1.427) | <0.001 | 1.163 (1.084, 1.247) | <0.001 |
| Others | 0.992 (0.932, 1.056) | 0.806 | 1.006 (0.938, 1.079) | 0.868 |
| Donor BMI | 0.993 (0.989, 0.996) | <0.001 | 0.999 (0.995, 1.003) | 0.555 |
| Donor Height | 0.995 (0.994, 0.997) | <0.001 | 0.997 (0.996, 0.999) | 0.002 |
| Donor Diabetes | 1.439 (1.337, 1.549) | <0.001 | 1.131 (1.040, 1.230) | 0.004 |
| Donor Hypertension | 1.185 (1.125, 1.247) | <0.001 | 1.061 (1.002, 1.123) | 0.042 |
| Donor Hep C Antibody, Positive | 1.213 (1.056, 1.394) | 0.006 | 1.185 (1.020, 1.378) | 0.027 |
| Donor Cause of Death (ref = “Other”) |  |  |  |  |
| Anoxia | 0.976 (0.853, 1.117) | 0.724 | 1.022 (0.877, 1.191) | 0.780 |
| Cerebrovascular/Stroke | 0.965 (0.845, 1.103) | 0.605 | 1.022 (0.878, 1.189) | 0.782 |
| Head Trauma | 0.917 (0.803, 1.047) | 0.202 | 0.987 (0.849, 1.148) | 0.870 |
| CNS Tumor | 0.874 (0.644, 1.187) | 0.388 | 0.858 (0.605, 1.218) | 0.392 |
| Donor Don. After Cardiac Death | 1.011 (0.945, 1.082) | 0.746 | 1.042 (0.969, 1.120) | 0.266 |
| Donor Terminal Creatinine | 1.026 (1.006, 1.046) | 0.010 | 1.015 (0.991, 1.040) | 0.231 |
| HLA antigen mismatch | 1.055 (1.041, 1.070) | <0.001 | 1.006 (0.992, 1.021) | 0.376 |
| Re-transplant | 1.011 (0.944, 1.083) | 0.760 | 1.187 (1.090, 1.292) | <0.001 |
| Transplant year (ref: 2001 – 2005) |  |  |  |  |
| 2006 - 2010 | 0.820 (0.777, 0.866) | <0.001 | 0.823 (0.773, 0.876) | <0.001 |
| 2011 - 2015 | 0.698 (0.659, 0.741) | <0.001 | 0.748 (0.701, 0.799) | <0.001 |
| Immunosuppression |  |  |  |  |
| T-cell Depletion Induction | 0.960 (0.902, 1.022) | 0.204 | 1.007 (0.937, 1.081) | 0.853 |
| IL-2RA | 0.938 (0.872, 1.009) | 0.084 | 1.073 (0.992, 1.160) | 0.078 |
| Calcineurin inhibitors | 0.815 (0.756, 0.878) | <0.001 | 0.818 (0.751, 0.891) | <0.001 |
| Steroids | 0.991 (0.936, 1.050) | 0.765 | 1.102 (1.035, 1.174) | 0.002 |

Abbreviations: AA, African American; BMI, Body mass index; CIT, Cold ischemia time; CNS, Central nervous system; LOS, Length of stay; TX, Transplant; IL-2RA, Interleukin-2 receptor antagonist; HLA, Human leukocyte antigen; PRA, Panel reactive antibody; Y, Yes

**Table S4.** Competing risk regression models for graft failure and patient death controlling all other covariates (living donors).

|  | Graft failure (frailty p-val < 0.001) | | Patient death (frailty p-val < 0.001) | |
| --- | --- | --- | --- | --- |
|  | HR (95% CI) | P-value | HR (95% CI) | P-value |
| Volume groups (ref = “Low”) |  |  |  |  |
| Medium | 0.991 (0.863, 1.137) | 0.897 | 1.021 (0.893, 1.167) | 0.762 |
| Medium-High | 0.937 (0.812, 1.081) | 0.370 | 0.912 (0.796, 1.045) | 0.183 |
| High | 0.960 (0.821, 1.123) | 0.613 | 0.891 (0.780, 1.019) | 0.092 |
| Recipient Age, Years | 0.966 (0.963, 0.969) | <0.001 | 1.055 (1.050, 1.059) | <0.001 |
| Recipient Sex, Male | 0.904 (0.831, 0.983) | 0.019 | 1.093 (0.986, 1.212) | 0.092 |
| Recipient Ethnicity (ref = “Caucasian”) |  |  |  |  |
| AA | 1.431 (1.203, 1.702) | <0.001 | 0.875 (0.683, 1.120) | 0.288 |
| Others | 0.908 (0.763, 1.081) | 0.278 | 0.563 (0.448, 0.706) | <0.001 |
| Recipient BMI | 1.013 (1.005, 1.021) | 0.001 | 0.995 (0.986, 1.004) | 0.298 |
| Recipient Diabetes, Y | 1.126 (1.019, 1.244) | 0.019 | 1.879 (1.701, 2.076) | <0.001 |
| Recipient Peak PRA | 1.003 (1.002, 1.005) | <0.001 | 1.001 (0.998, 1.003) | 0.522 |
| Recipient Kidney CIT | 0.999 (0.991, 1.007) | 0.816 | 1.004 (0.995, 1.013) | 0.373 |
| Recipient Hepatitis C Antibody, Positive | 1.681 (1.353, 2.088) | <0.001 | 1.615 (1.279, 2.040) | <0.001 |
| Recipient Dialysis at TX, Y | 1.517 (1.368, 1.681) | <0.001 | 1.597 (1.426, 1.790) | <0.001 |
| Recipient LOS, Days | 1.003 (0.999, 1.006) | 0.097 | 1.004 (1.002, 1.005) | <0.001 |
| Treated for rejection within 1 year (kidney) | 2.851 (2.587, 3.140) | <0.001 | 1.333 (1.142, 1.554) | <0.001 |
| Donor Age, Years | 1.019 (1.015, 1.023) | <0.001 | 1.005 (1.001, 1.010) | 0.020 |
| Donor Sex, Male | 0.980 (0.871, 1.102) | 0.731 | 1.026 (0.894, 1.177) | 0.717 |
| Donor Ethnicity (ref = “Caucasian”) |  |  |  |  |
| AA | 1.411 (1.177, 1.692) | <0.001 | 1.018 (0.784, 1.323) | 0.893 |
| Others | 0.977 (0.817, 1.168) | 0.795 | 0.929 (0.74, 1.1680) | 0.530 |
| Donor BMI | 0.996 (0.987, 1.006) | 0.437 | 1.012 (1.001, 1.024) | 0.028 |
| Donor Height | 0.989 (0.983, 0.995) | <0.001 | 0.997 (0.990, 1.003) | 0.324 |
| Donor Hypertension | 1.189 (0.931, 1.519) | 0.166 | 1.149 (0.887, 1.488) | 0.294 |
| HLA antigen mismatch | 1.073 (1.046, 1.101) | <0.001 | 1.007 (0.977, 1.037) | 0.672 |
| Re-transplant | 1.109 (0.967, 1.271) | 0.138 | 1.329 (1.117, 1.581) | 0.001 |
| Transplant year (ref: 2001 – 2005) |  |  |  |  |
| 2006 – 2010 | 0.797 (0.714, 0.890) | <0.001 | 0.820 (0.716, 0.938) | 0.004 |
| 2011 – 2015 | 0.661 (0.591, 0.740) | <0.001 | 0.778 (0.681, 0.888) | <0.001 |
| Immunosuppression |  |  |  |  |
| T-cell Depletion Induction | 1.040 (0.921, 1.174) | 0.531 | 1.003 (0.875, 1.150) | 0.963 |
| IL-2RA | 0.973 (0.855, 1.108) | 0.683 | 1.012 (0.879, 1.166) | 0.868 |
| Calcineurin inhibitors | 0.852 (0.738, 0.984) | 0.030 | 0.794 (0.674, 0.934) | 0.006 |
| Steroids | 0.920 (0.830, 1.021) | 0.116 | 1.054 (0.944, 1.178) | 0.348 |

Abbreviations: AA, African American; BMI, Body mass index; CIT, Cold ischemia time; LOS, Length of stay; TX, Transplant; IL-2RA, Interleukin-2 receptor antagonist; HLA, Human leukocyte antigen; PRA, Panel reactive antibody; Y, Yes

**Table S5.** Competing risk regression models for graft failure and patient death controlling all other covariates (diabetic patients of living donors).

|  | Graft failure (frailty p-val < 0.001) | | Patient death (frailty p-val < 0.001) | |
| --- | --- | --- | --- | --- |
|  | HR (95% CI) | P-value | HR (95% CI) | P-value |
| Volume groups (ref = “Low”) |  |  |  |  |
| Medium | 1.087 (0.847, 1.395) | 0.514 | 1.001 (0.830, 1.209) | 0.989 |
| Medium-High | 0.880 (0.677, 1.145) | 0.342 | 0.824 (0.677, 1.003) | 0.053 |
| High | 0.838 (0.637, 1.103) | 0.207 | 0.887 (0.735, 1.070) | 0.209 |
| Recipient Age, Years | 0.975 (0.968, 0.983) | <0.001 | 1.040 (1.033, 1.048) | <0.001 |
| Recipient Sex, Male | 0.824 (0.693, 0.980) | 0.029 | 0.976 (0.840, 1.135) | 0.755 |
| Recipient Ethnicity (ref = “Caucasian”) |  |  |  |  |
| AA | 1.485 (1.041, 2.118) | 0.029 | 0.917 (0.649, 1.296) | 0.624 |
| Others | 1.140 (0.833, 1.558) | 0.413 | 0.597 (0.444, 0.803) | 0.001 |
| Recipient BMI | 1.019 (1.005, 1.035) | 0.010 | 0.999 (0.986, 1.013) | 0.936 |
| Recipient Peak PRA | 1.004 (1.000, 1.007) | 0.043 | 1.002 (0.999, 1.006) | 0.152 |
| Recipient Kidney CIT | 1.002 (0.983, 1.023) | 0.808 | 1.011 (0.998, 1.024) | 0.105 |
| Recipient Hepatitis C Antibody, Positive | 1.858 (1.293, 2.670) | 0.001 | 1.034 (0.701, 1.523) | 0.867 |
| Recipient Dialysis at TX, Y | 1.317 (1.070, 1.620) | 0.009 | 1.299 (1.102, 1.530) | 0.002 |
| Recipient LOS, Days | 1.010 (1.002, 1.018) | 0.016 | 1.013 (1.008, 1.018) | <0.001 |
| Treated for rejection within 1 year (kidney) | 2.859 (2.337, 3.498) | <0.001 | 1.033 (0.806, 1.323) | 0.799 |
| Donor Age, Years | 1.020 (1.012, 1.028) | <0.001 | 1.004 (0.998, 1.010) | 0.239 |
| Donor Sex, Male | 0.979 (0.773, 1.240) | 0.863 | 1.126 (0.927, 1.368) | 0.233 |
| Donor Ethnicity (ref = “Caucasian”) |  |  |  |  |
| AA | 1.015 (0.696, 1.480) | 0.939 | 0.899 (0.622, 1.301) | 0.574 |
| Others | 0.915 (0.656, 1.276) | 0.599 | 0.924 (0.680, 1.256) | 0.614 |
| Donor BMI | 1.008 (0.990, 1.027) | 0.376 | 1.020 (1.004, 1.036) | 0.013 |
| Donor Height | 0.986 (0.975, 0.998) | 0.021 | 0.994 (0.984, 1.003) | 0.207 |
| Donor Hypertension | 1.079 (0.690, 1.689) | 0.738 | 0.935 (0.632, 1.385) | 0.738 |
| HLA antigen mismatch | 1.073 (1.018, 1.130) | 0.009 | 1.029 (0.985, 1.075) | 0.204 |
| Re-transplant | 0.921 (0.660, 1.287) | 0.631 | 1.033 (0.775, 1.378) | 0.825 |
| Transplant year (ref: 2001 – 2005) |  |  |  |  |
| 2006 – 2010 | 0.812 (0.652, 1.010) | 0.062 | 0.874 (0.718, 1.065) | 0.182 |
| 2011 – 2015 | 0.629 (0.501, 0.790) | <0.001 | 0.871 (0.718, 1.058) | 0.164 |
| Immunosuppression |  |  |  |  |
| T-cell Depletion Induction | 0.958 (0.760, 1.209) | 0.719 | 1.064 (0.872, 1.297) | 0.542 |
| IL-2RA | 0.831 (0.648, 1.067) | 0.146 | 1.086 (0.884, 1.334) | 0.430 |
| Calcineurin inhibitors | 0.726 (0.552, 0.956) | 0.022 | 0.778 (0.615, 0.983) | 0.036 |
| Steroids | 0.992 (0.812, 1.212) | 0.936 | 1.139 (0.973, 1.334) | 0.105 |

Abbreviations: AA, African American; BMI, Body mass index; CIT, Cold ischemia time; LOS, Length of stay; TX, Transplant, IL-2RA, Interleukin-2 receptor antagonist; PRA, Panel reactive antibody; HLA, Human leukocyte antigen; Y, Yes

**Table S6.** Competing risk regression models for graft failure and patient death controlling all other covariates (diabetic patients of deceased donors).

|  | Graft failure (frailty p-val < 0.001) | | Patient death (frailty p-val < 0.001) | |
| --- | --- | --- | --- | --- |
|  | HR (95% CI) | P-value | HR (95% CI) | P-value |
| Volume groups (ref = “Low”) |  |  |  |  |
| Medium | 0.976 (0.870, 1.096) | 0.686 | 0.967 (0.869, 1.076) | 0.537 |
| Medium-High | 0.867 (0.766, 0.981) | 0.024 | 0.858 (0.763, 0.965) | 0.011 |
| High | 0.897 (0.774, 1.038) | 0.144 | 0.957 (0.833, 1.099) | 0.531 |
| Recipient Age, Years | 0.983 (0.979, 0.987) | <0.001 | 1.043 (1.039, 1.046) | <0.001 |
| Recipient Sex, Male | 0.925 (0.855, 1.002) | 0.055 | 1.079 (1.005, 1.158) | 0.036 |
| Recipient Ethnicity (ref = “Caucasian”) |  |  |  |  |
| AA | 1.308 (1.197, 1.430) | <0.001 | 0.692 (0.639, 0.750) | <0.001 |
| Others | 0.903 (0.811, 1.006) | 0.064 | 0.611 (0.558, 0.668) | <0.001 |
| Recipient BMI | 1.013 (1.006, 1.020) | <0.001 | 0.991 (0.984, 0.997) | 0.004 |
| Recipient Peak PRA | 1.002 (1.000, 1.003) | 0.011 | 1.000 (0.999, 1.002) | 0.6 |
| Recipient Kidney CIT | 1.007 (1.002, 1.011) | 0.003 | 1.003 (0.999, 1.007) | 0.213 |
| Recipient Hepatitis C Antibody, Positive | 1.203 (1.011, 1.430) | 0.037 | 1.280 (1.097, 1.493) | 0.002 |
| Recipient Dialysis at TX, Y | 1.237 (1.060, 1.444) | 0.007 | 1.525 (1.337, 1.740) | <0.001 |
| Recipient LOS, Days | 1.005 (1.003, 1.007) | <0.001 | 1.005 (1.004, 1.007) | <0.001 |
| Treated for rejection within 1 year (kidney) | 2.615 (2.386, 2.865) | <0.001 | 1.327 (1.196, 1.472) | <0.001 |
| Donor Age, Years | 1.020 (1.017, 1.023) | <0.001 | 1.003 (1.001, 1.006) | 0.014 |
| Donor Sex, Male | 1.060 (0.972, 1.155) | 0.187 | 1.086 (1.008, 1.170) | 0.031 |
| Donor Ethnicity (ref = “Caucasian”) |  |  |  |  |
| AA | 1.472 (1.331, 1.627) | <0.001 | 1.153 (1.046, 1.272) | 0.004 |
| Others | 0.935 (0.835, 1.048) | 0.248 | 0.989 (0.898, 1.090) | 0.824 |
| Donor BMI | 0.993 (0.987, 0.999) | 0.015 | 0.997 (0.992, 1.002) | 0.223 |
| Donor Height | 0.990 (0.988, 0.993) | <0.001 | 0.997 (0.995, 1.000) | 0.039 |
| Donor Diabetes | 1.414 (1.255, 1.593) | <0.001 | 1.136 (1.012, 1.276) | 0.031 |
| Donor Hypertension | 1.261 (1.155, 1.376) | <0.001 | 1.120 (1.035, 1.211) | 0.005 |
| Donor Hep C Antibody, Positive | 1.275 (1.013, 1.604) | 0.038 | 1.213 (0.985, 1.492) | 0.069 |
| Donor Cause of Death (ref = “Other”) | 0.949 (0.750, 1.201) | 0.663 | 1.127 (0.908, 1.399) | 0.276 |
| Anoxia | 0.890 (0.706, 1.121) | 0.322 | 1.102 (0.890, 1.366) | 0.372 |
| Cerebrovascular/Stroke | 0.869 (0.688, 1.096) | 0.236 | 1.030 (0.831, 1.275) | 0.789 |
| Head Trauma | 0.745 (0.421, 1.317) | 0.311 | 1.067 (0.663, 1.719) | 0.788 |
| CNS Tumor | 1.012 (0.899, 1.140) | 0.839 | 1.056 (0.956, 1.166) | 0.285 |
| Donor Don. After Cardiac Death | 1.031 (0.992, 1.072) | 0.118 | 1.028 (0.993, 1.063) | 0.115 |
| Donor Terminal Creatinine | 1.070 (1.044, 1.097) | <0.001 | 1.018 (0.998, 1.038) | 0.083 |
| HLA antigen mismatch | 1.065 (0.917, 1.236) | 0.412 | 1.068 (0.930, 1.225) | 0.351 |
| Re-transplant |  |  |  |  |
| Transplant year (ref: 2001 – 2005) |  |  |  |  |
| 2006 – 2010 | 0.823 (0.745, 0.910) | <0.001 | 0.820 (0.751, 0.896) | <0.001 |
| 2011 – 2015 | 0.678 (0.611, 0.753) | <0.001 | 0.726 (0.662, 0.795) | <0.001 |
| Immunosuppression |  |  |  |  |
| T-cell Depletion Induction | 1.073 (0.964, 1.195) | 0.198 | 1.017 (0.923, 1.120) | 0.735 |
| IL-2RA Induction | 1.090 (0.964, 1.233) | 0.170 | 1.073 (0.965, 1.193) | 0.194 |
| Calcineurin Inhibitor | 0.788 (0.689, 0.901) | 0.001 | 0.850 (0.753, 0.960) | 0.009 |
| Maintenance Steroids | 0.977 (0.89, 1.073) | 0.632 | 1.121 (1.031, 1.22) | 0.008 |

Abbreviations: AA, African American; BMI, Body mass index; CIT, Cold ischemia time; CNS, Central nervous system; LOS, Length of stay; TX, Transplant, IL-2RA, Interleukin-2 receptor antagonist; PRA, Panel reactive antibody; HLA, Human leukocyte antigen; Y, Yes

**Table S7.** Competing risk regression models for graft failure and patient death controlling all other covariates (KDPI>85% donors).

|  | Graft failure (frailty p-val < 0.001) | | Patient death (frailty p-val < 0.001) | |
| --- | --- | --- | --- | --- |
|  | HR (95% CI) | P-value | HR (95% CI) | P-value |
| Volume groups (ref = “Low”) |  |  |  |  |
| Medium | 1.129 (0.927, 1.374) | 0.228 | 1.014 (0.826, 1.245) | 0.897 |
| Medium-High | 1.042 (0.854, 1.273) | 0.683 | 0.840 (0.678, 1.040) | 0.11 |
| High | 1.04 (0.825, 1.31) | 0.741 | 0.829 (0.646, 1.064) | 0.141 |
| Recipient Age, Years | 0.989 (0.982, 0.995) | 0.001 | 1.060 (1.051, 1.070) | <0.001 |
| Recipient Sex, Male | 1.217 (1.062, 1.395) | 0.005 | 1.010 (0.873, 1.168) | 0.896 |
| Recipient Ethnicity (ref = “Caucasian”) |  |  |  |  |
| AA | 1.278 (1.100, 1.484) | 0.001 | 0.621 (0.521, 0.740) | <0.001 |
| Others | 0.872 (0.721, 1.055) | 0.159 | 0.677 (0.559, 0.820) | <0.001 |
| Recipient BMI | 1.016 (1.004, 1.029) | 0.012 | 0.986 (0.972, 1.001) | 0.065 |
| Recipient Diabetes, Y | 1.208 (1.060, 1.377) | 0.005 | 1.610 (1.395, 1.858) | <0.001 |
| Recipient Peak PRA | 1.002 (0.999, 1.004) | 0.322 | 1.003 (0.999, 1.006) | 0.144 |
| Recipient Kidney CIT | 1.005 (0.997, 1.012) | 0.223 | 0.998 (0.989, 1.006) | 0.559 |
| Recipient Hepatitis C Antibody, Positive | 1.060 (0.785, 1.433) | 0.703 | 1.415 (0.994, 2.015) | 0.054 |
| Recipient Dialysis at TX, Y | 1.355 (1.052, 1.744) | 0.019 | 1.212 (0.959, 1.532) | 0.108 |
| Recipient LOS, Days | 1.004 (0.999, 1.009) | 0.096 | 1.005 (1.001, 1.009) | 0.016 |
| Treated for rejection within 1 year (kidney) | 2.467 (2.124, 2.864) | <0.001 | 1.494 (1.228, 1.817) | <0.001 |
| Donor Age, Years | 1.024 (1.013, 1.035) | <0.001 | 1.001 (0.989, 1.013) | 0.844 |
| Donor Sex, Male | 0.937 (0.801, 1.096) | 0.413 | 0.937 (0.792, 1.110) | 0.454 |
| Donor Ethnicity (ref = “Caucasian”) |  |  |  |  |
| AA | 1.299 (1.107, 1.523) | 0.001 | 1.158 (0.965, 1.390) | 0.115 |
| Others | 1.110 (0.909, 1.355) | 0.307 | 1.067 (0.858, 1.327) | 0.560 |
| Donor BMI | 0.997 (0.987, 1.007) | 0.527 | 0.999 (0.989, 1.010) | 0.895 |
| Donor Height | 0.995 (0.988, 1.002) | 0.162 | 1.001 (0.994, 1.009) | 0.745 |
| Donor Diabetes | 1.233 (1.060, 1.434) | 0.007 | 1.038 (0.876, 1.229) | 0.669 |
| Donor Hypertension | 1.138 (0.963, 1.345) | 0.13 | 1.231 (1.023, 1.483) | 0.028 |
| Donor Hep C Antibody, Positive | 1.830 (1.284, 2.609) | 0.001 | 1.111 (0.704, 1.752) | 0.652 |
| Donor Cause of Death (ref = “Other”) |  |  |  |  |
| Anoxia | 1.731 (0.801, 3.743) | 0.163 | 1.128 (0.607, 2.095) | 0.704 |
| Cerebrovascular/Stroke | 1.437 (0.676, 3.058) | 0.346 | 0.935 (0.515, 1.697) | 0.825 |
| Head Trauma | 1.439 (0.657, 3.152) | 0.363 | 0.943 (0.500, 1.780) | 0.856 |
| CNS Tumor | 0.716 (0.087, 5.878) | 0.756 | - | - |
| Donor Don. After Cardiac Death | 1.035 (0.793, 1.353) | 0.798 | 1.156 (0.884, 1.511) | 0.290 |
| Donor Terminal Creatinine | 1.016 (0.965, 1.071) | 0.542 | 1.040 (0.983, 1.101) | 0.175 |
| HLA antigen mismatch | 1.030 (0.980, 1.083) | 0.239 | 1.010 (0.959, 1.065) | 0.700 |
| Re-transplant | 1.069 (0.807, 1.416) | 0.642 | 1.332 (0.963, 1.842) | 0.083 |
| Transplant year (ref: 2001 – 2005) |  |  |  |  |
| 2006 - 2010 | 0.845 (0.717, 0.996) | 0.044 | 0.768 (0.637, 0.925) | 0.005 |
| 2011 - 2015 | 0.694 (0.579, 0.831) | <0.001 | 0.693 (0.570, 0.842) | <0.001 |
| Immunosuppression |  |  |  |  |
| T-cell Depletion Induction | 0.987 (0.830, 1.173) | 0.882 | 1.272 (1.025, 1.578) | 0.029 |
| IL-2RA Induction | 0.896 (0.728, 1.104) | 0.304 | 1.292 (1.016, 1.642) | 0.037 |
| Calcineurin Inhibitor | 0.897 (0.716, 1.123) | 0.342 | 0.826 (0.642, 1.063) | 0.138 |
| Maintenance Steroids | 1.077 (0.924, 1.256) | 0.343 | 1.150 (0.970, 1.363) | 0.107 |

Abbreviations: AA, African American; BMI, Body mass index; CIT, Cold ischemia time; CNS, Central nervous system; LOS, Length of stay; TX, Transplant, IL-2RA, Interleukin-2 receptor antagonist; PRA, Panel reactive antibody; HLA, Human leukocyte antigen; Y, Yes

**Table S8.** Competing risk regression models for graft failure and patient death controlling all other covariates (kidney re-transplant patients-deceased donors).

|  | Graft failure (frailty p-val < 0.001) | | Patient death (frailty p-val < 0.001) | |
| --- | --- | --- | --- | --- |
|  | HR (95% CI) | P-value | HR (95% CI) | P-value |
| Volume groups (ref = “Low”) |  |  |  |  |
| Medium | 1.076 (0.908, 1.275) | 0.400 | 0.999 (0.820, 1.218) | 0.992 |
| Medium-High | 1.005 (0.840, 1.202) | 0.959 | 0.958 (0.784, 1.172) | 0.679 |
| High | 1.136 (0.934, 1.382) | 0.203 | 0.971 (0.784, 1.203) | 0.789 |
| Recipient Age, Years | 0.973 (0.968, 0.977) | <0.001 | 1.052 (1.046, 1.059) | <0.001 |
| Recipient Sex, Male | 0.990 (0.886, 1.106) | 0.859 | 1.199 (1.031, 1.394) | 0.018 |
| Recipient Ethnicity (ref = “Caucasian”) |  |  |  |  |
| AA | 1.125 (0.987, 1.282) | 0.077 | 0.853 (0.712, 1.021) | 0.083 |
| Others | 0.872 (0.735, 1.034) | 0.115 | 0.528 (0.409, 0.681) | <0.001 |
| Recipient BMI | 1.023 (1.013, 1.033) | <0.001 | 0.998 (0.983, 1.012) | 0.737 |
| Recipient Diabetes, Y | 1.051 (0.905, 1.220) | 0.512 | 1.560 (1.336, 1.823) | <0.001 |
| Recipient Peak PRA | 1.002 (1.000, 1.003) | 0.027 | 0.999 (0.997, 1.001) | 0.389 |
| Recipient Kidney CIT | 1.003 (0.996, 1.01) | 0.474 | 1.008 (1.000, 1.017) | 0.061 |
| Recipient Hepatitis C Antibody, Positive | 1.575 (1.283, 1.934) | <0.001 | 1.546 (1.176, 2.031) | 0.002 |
| Recipient Dialysis at TX, Y | 1.445 (1.151, 1.816) | 0.002 | 1.435 (1.112, 1.851) | 0.005 |
| Recipient LOS, Days | 1.003 (1.000, 1.007) | 0.080 | 1.008 (1.005, 1.011) | <0.001 |
| Treated for rejection within 1 year (kidney) | 2.824 (2.501, 3.188) | <0.001 | 1.359 (1.103, 1.676) | 0.004 |
| Donor Age, Years | 1.015 (1.011, 1.020) | <0.001 | 1.004 (0.998, 1.010) | 0.229 |
| Donor Sex, Male | 1.002 (0.881, 1.139) | 0.980 | 1.104 (0.932, 1.306) | 0.252 |
| Donor Ethnicity (ref = “Caucasian”) |  |  |  |  |
| AA | 1.419 (1.216, 1.654) | <0.001 | 1.048 (0.829, 1.325) | 0.693 |
| Others | 0.892 (0.752, 1.058) | 0.190 | 0.814 (0.646, 1.027) | 0.082 |
| Donor BMI | 0.992 (0.984, 1.001) | 0.094 | 1.006 (0.994, 1.018) | 0.313 |
| Donor Height | 0.993 (0.989, 0.997) | 0.002 | 0.995 (0.990, 1.001) | 0.092 |
| Donor Diabetes | 1.576 (1.273, 1.951) | <0.001 | 1.150 (0.858, 1.541) | 0.349 |
| Donor Hypertension | 1.068 (0.924, 1.234) | 0.375 | 0.975 (0.803, 1.185) | 0.802 |
| Donor Hep C Antibody, Positive | 0.703 (0.437, 1.130) | 0.145 | 0.952 (0.590, 1.536) | 0.839 |
| Donor Cause of Death (ref = “Other”) |  |  |  |  |
| Anoxia | 1.190 (0.783, 1.810) | 0.415 | 1.342 (0.791, 2.275) | 0.276 |
| Cerebrovascular/Stroke | 1.337 (0.881, 2.029) | 0.173 | 1.403 (0.828, 2.376) | 0.208 |
| Head Trauma | 1.186 (0.785, 1.793) | 0.418 | 1.100 (0.652, 1.857) | 0.720 |
| CNS Tumor | 1.610 (0.763, 3.396) | 0.211 | 1.308 (0.472, 3.622) | 0.606 |
| Donor Don. After Cardiac Death | 1.090 (0.910, 1.306) | 0.349 | 1.049 (0.837, 1.314) | 0.678 |
| Donor Terminal Creatinine | 1.076 (1.036, 1.118) | <0.001 | 0.966 (0.877, 1.063) | 0.480 |
| HLA antigen mismatch | 1.038 (1.006, 1.071) | 0.020 | 0.983 (0.944, 1.023) | 0.394 |
| Transplant year (ref: 2001 – 2005) |  |  |  |  |
| 2006 - 2010 | 0.725 (0.627, 0.839) | <0.001 | 0.829 (0.678, 1.013) | 0.067 |
| 2011 - 2015 | 0.662 (0.568, 0.773) | <0.001 | 0.791 (0.644, 0.973) | 0.026 |
| Immunosuppression |  |  |  |  |
| T-cell Depletion Induction | 0.841 (0.719, 0.983) | 0.030 | 1.164 (0.934, 1.450) | 0.176 |
| IL-2RA Induction | 0.774 (0.621, 0.964) | 0.022 | 1.431 (1.097, 1.866) | 0.008 |
| Calcineurin Inhibitor | 0.828 (0.680, 1.007) | 0.059 | 0.646 (0.511, 0.815) | <0.001 |
| Maintenance Steroids | 0.934 (0.805, 1.084) | 0.372 | 1.074 (0.888, 1.298) | 0.461 |

Abbreviations: AA, African American; BMI, Body mass index; CIT, Cold ischemia time; CNS, Central nervous system; LOS, Length of stay; TX, Transplant, IL-2RA, Interleukin-2 receptor antagonist; PRA, Panel reactive antibody; HLA, Human leukocyte antigen; Y, Yes
