# Supplementary material for: "Long-term effects of center volume on transplant outcomes in adult kidney transplant recipients"
Source: PLoS One. 2024 Jun 6;19(6):e0301425. doi: 10.1371/journal.pone.0301425 (PMC11156332; doi:10.1371/journal.pone.0301425)
Supplement: Attachment — Submitted filename: PONE-D-23-11616_review_MM_20230730.docx [file pone.0301425.s002.docx]

This is a nationwide cohort study of adult kidney transplant recipients to estimate the association between kidney transplant center volume and 5-year outcomes. The authors reported that medium-high center volume was associated with improved patient and graft survival in deceased donor kidney transplant recipients. Although this is an interesting study, there are several concerns that need to be addressed.

**Title**

1) Based on the STROBE statement, I would suggest that the authors describe the study’s design in the title. The study’s design is more important than the statistical analysis.

**Abstract**

2) I would suggest that the authors ensure consistency in the methods, materials, and conclusions. The abstract should describe the results for the entire cohort, i.e., both living and deceased donor kidney transplant recipients, rather than only deceased donor kidney transplant recipients.

**Introduction**

3) I would suggest that the focus be on long-term follow-up as well as novelty of statistical analysis. Considering the similar previous studies, the novelty of this study lies more in the long-term follow-up than in the statistical analysis.
4) The authors stated that "We hypothesized that center volume will affect the clinical outcomes including graft failure and patient mortality." I would suggest that the authors provide a rationale for their hypothesis based on the results of the previous studies. Do the authors believe that the negative results of previous studies were due to inappropriate statistical analysis, i.e., the Cox proportional hazards model? As I mentioned above, please consider restructuring the introduction section to better clarify the novelty of the study and the rationale for the hypothesis.

**Materials and Methods**

5) I would suggest clarifying that the study population was "kidney-only" transplant recipients.
6) I would suggest that "Exposure" and "Outcomes" be described as separate sections.

**Statistical analysis**

7) I would suggest that the authors reconsider the statistical analysis, especially the subgroup analysis of kidney transplant recipients with diabetes. I understood that recipients of living and deceased donor organs were analyzed separately because of the different predicted graft longevity and because of the different covariate adjustment. This statistical approach was conducted in the previous study [1]. However, for the analysis of kidney transplant recipients with diabetes, both living and deceased donors seemed to be included in this study (Table S5). If the authors do not distinguish such recipients, then the recipients of the entire cohort should be included in the main analysis.

[1] Sonnenberg EM, Cohen JB, Hsu JY, Potluri VS, Levine MH, Abt PL, et al. Association of kidney transplant center volume with 3-year clinical outcomes. Am J Kidney Dis. 2019; 74(4):441–451.

8) I would suggest clarifying the handling of missing data. Given the results of the previous study using Organ Procurement and Transplantation Network registry data [1], I assumed that there were missing data in this study as well. Did you perform a complete case analysis? I would suggest performing the same analysis after multiple imputation of all missing covariates as a sensitivity analysis.

[1] Sonnenberg EM, Cohen JB, Hsu JY, Potluri VS, Levine MH, Abt PL, et al. Association of kidney transplant center volume with 3-year clinical outcomes. Am J Kidney Dis. 2019; 74(4):441–451.

9) Please consider restructuring the statistical analysis section to better clarify its methodology. I understood that the authors conducted competing risk analyses because the authors stated in the introduction section that "Their results show that CRA may provide more accurate estimates of long-term graft survival and death, particularly in subgroups of recipients with higher rates of competing events". In addition, the authors specified "competing risk analysis" in their title. On the other hand, the authors also stated in the statistical analysis section that "We fitted our cause-specific hazards model using the “coxph” function in the “survival” package in R." I would propose to estimate both the cause-specific hazard ratios and the subdistribution hazard ratios.
10) Given that patients who received kidney transplantation between January 2001 and December 2015 were included in this study, an adjustment by year of transplant may be necessary. In addition to immunosuppressive drugs, the quality of transplant team care and surgical techniques has evolved over time.

**Results**

11) When using competing risk analysis, I would suggest that the adjusted "subdistribution hazard ratio" be stated instead of the adjusted "hazard ratio" throughout the manuscript to avoid confusing the readers.

**Discussion**

12) I would propose to revise the text regarding the summary of results in the first paragraph of discussion section as follows: we report that, compared to low (Q1) volume centers, medium-high (Q3) volume (median: 121 transplants/year) US transplant centers had better outcomes. The Q1 center volume was set as the reference throughout the analysis.
13) I would suggest that the authors further discuss the differences in outcomes between living and deceased donor kidney transplantation. Why was kidney transplant center volume not associated with both graft loss and mortality in living donor kidney transplant recipients? Could this discrepancy be due to differences in the graft survival of living and deceased donor kidney transplant recipients?

**Figure**

14) In the Kaplan-Meier curve, I would suggest that the number of recipients in each group be listed at the bottom of the curve.

**Table**

15) I would suggest that the number of recipients in each group be listed at the top of the table. For example, Low (Q1) (n = ????).
16) As I mentioned above, I would suggest that the results of the competing analysis and the Cox proportional hazards model be described side by side. I am interested in whether the results of the two differ from each other.
