# Supplementary material for: "Long-term effects of center volume on transplant outcomes in adult kidney transplant recipients"
Source: PLoS One. 2024 Jun 6;19(6):e0301425. doi: 10.1371/journal.pone.0301425 (PMC11156332; doi:10.1371/journal.pone.0301425)
Supplement: Attachment — Submitted filename: Response to reviewers PLOSONE.docx [file pone.0301425.s003.docx]

Response to the reviewers:

Responses to the comments are in blue.

Changes in the manuscript are in red.

**Reviewer’s Comments:**

**Reviewer #1 Comments:**

In this UNOS database analysis using a competing risk model, the authors compared Kdiney transplant outcomes (5-year graft failure and patient death) based on center volume (categorized as low, medium, medium-high and high volume centers). The study found best outcome for deceased donor kidney recipients, diabetic recipients and those who received kidneys with KDPI >85% for transplants performed in medium-high volume transplant centers. The article is written well and the analysis is detailed.

Thank you for your thorough review of our UNOS database analysis. We appreciate your positive comments on the well-written article and detailed analysis. Your feedback on the outcomes based on center volume is valuable. We will consider your suggestions for improvement.

1. Factors such as dialysis duration before transplant, and delayed graft function after transplant can impact long term graft and patient outcomes that should be accounted for in the analysis to minimize residual bias.

Thank you for your valuable feedback. Regarding delayed graft function (DGF), we chose not to include it in our analysis due to the variability in definitions. To explore this aspect further, please refer to the information available in Center-Level Variation in the Development of Delayed Graft Function Following Deceased Donor Kidney Transplantation - PMC (nih.gov).

https://www.ncbi.nlm.nih.gov/pmc/articles/PMC4405384/

2. Why was death-censored graft failure not calculated that would censor for death with functioning graft?

Thanks for your inquiry. In our competing risk analysis, we intentionally did not calculate death-censored graft failure. Given the nature of our study, where graft failure and death are competing factors, incorporating death-censored graft failure wouldn't offer additional insights.

3. Type of health insurance, and socioeconomic status reflected in median household income can all impact post-transplant outcomes. An attempt should be made to incorporate these variables in the analysis.

Thank you for your valuable input. While we appreciate the significance of variables like health insurance type and socio-economic status (SES) in post-transplant outcomes, we decided not to include them in our analysis due to their unreliability. It's noteworthy that Sonnenberg did not incorporate these variables, and the introduction of the Affordable Care Act has led to changes in coverage over the years, introducing additional complexity. Moreover, the fluctuating nature of state Medicaid coverage rules and the potential for insurance changes after listing render these variables less reliable for our study. We appreciate your understanding of these considerations.

4. It would be interesting to know the transplant rate (per hundred person years) between the groups. If transplant rate is low, it could be an indication that those centers were more selective in using only better-quality kidneys which can translate into better outcomes.

Thank you for the clarification. It appears you have used annual rates in Table 1 to describe the center volume dynamics, specifically providing insights into low-volume centers. Utilizing annual rates can indeed offer valuable information on the transplantation activities of each center over the study period.

**Reviewer #2 Comments:**

This is a nationwide cohort study of adult kidney transplant recipients to estimate the association between kidney transplant center volume and 5-year outcomes. The authors reported that medium-high center volume was associated with improved patient and graft survival in deceased donor kidney transplant recipients. Although this is an interesting study, several concerns need to be addressed.

We value the reviewer's insightful assessment of our nationwide cohort study examining kidney transplant outcomes. The raised concerns have been duly noted, and we are fully committed to thoroughly addressing them. Your feedback is highly appreciated, and we are determined to enhance the quality and precision of our study to meet rigorous scientific standards.

**Title:**

**1)**Based on the STROBE statement, I would suggest that the authors describe the study’s design in the title. The study’s design is more important than the statistical analysis.

Thank you for your insightful feedback. We appreciate your suggestion, and in response, we have revised the title of our manuscript to better align with the emphasis on long-term outcomes. The updated title now reads: "Long-Term Effects of Center Volume on Transplant Outcomes in Adult Kidney Transplant Recipients."

We believe this modification enhances the clarity of our study's focus on long-term implications. We value your guidance and hope that this adjustment aligns with your expectations.

**Abstract**

**2)**I would suggest that the authors ensure consistency in the methods, materials, and conclusions. The abstract should describe the results for the entire cohort, i.e., both living and deceased donor kidney transplant recipients, rather than only deceased donor kidney transplant recipients.

Thank you for your feedback. We have revised the abstract to ensure consistency by describing results for both living and deceased donor kidney transplant recipients as below. This modification enhances the overall comprehensiveness of our findings. We appreciate your guidance and believe that this adjustment aligns with your expectations.

**Background:** The influence of center volume on kidney transplant outcomes is a topic of ongoing debate. In this study, we employed competing risk analyses to accurately estimate the marginal probability of graft failure in the presence of competing events, such as mortality from other causes with long-term outcomes. The incorporation of immunosuppression protocols and extended follow-up offers additional insights. Our emphasis on long-term follow-up aligns with biological considerations where competing risks play a significant role.

**Methods:** We examined data from 219,878 adult kidney only transplantations across 256 U.S. transplant centers (January 2001-December 2015) sourced from the Organ Procurement and Transplantation Network registry. Centers were classified into quartiles by annual volume: low (Q1=28), medium (Q2=75), medium-high (Q3=121), and high (Q4=195). Our study investigated the relationship between center volume and 5-year outcomes, focusing on graft failure and mortality. Sub-population analyses included deceased donor, living donor, diabetic recipients, those with kidney donor profile index >85%, and re-transplants from deceased donors.

**Results:**

Adjusted cause-specific hazard ratios (aCHR) for Five-Year Graft Failure and Patient Death were examined by center volume, with low-volume centers as the reference standard (aCHR: 1.0). In deceased donors, medium-high and high-volume centers showed significantly lower cause-specific hazard ratios for graft failure (medium-high aCHR=0.892, p<0.001; high aCHR=0.953, p=0.149) and patient death (medium-high aCHR=0.828, p<0.001; high aCHR=0.898, p=0.003). Among living donors, no significant differences were found for graft failure, while a trend towards lower cause-specific hazard ratios for patient death was observed in medium-high (aCHR=0.895, p=0.107) and high-volume centers (aCHR=0.88, p=0.061).

**Conclusion:** Higher center volume is associated with significantly lower cause-specific hazard ratios for graft failure and patient death in deceased donors, while a trend towards reduced cause-specific hazard ratios for patient death is observed in living donors.

**Introduction**

**3)**I would suggest that the focus be on long-term follow-up as well as novelty of statistical analysis. Considering the similar previous studies, the novelty of this study lies more in the long-term follow-up than in the statistical analysis.

We appreciate the reviewer's valuable feedback. In response, we have refocused the manuscript to underscore the significance of long-term follow-up in conjunction with the novelty of the statistical analysis, specifically the competing risk analysis (CRA) technique. The revised manuscript highlights how CRA is adept at capturing subtle differences, especially over an extended 5-year follow-up period. This adjustment aims to emphasize the dual importance of both long-term observation and the innovative statistical approach in our study, addressing the reviewer's suggestion.

Introduction page 4,5:

Sonnenberg et al categorized kidney transplant centers into low, medium, medium-high, and high volume based on the mean annual number of kidney transplants from 2009 to 2013. Their findings indicated no evidence of improved outcomes with increased center volume^8^. The study's limitations, including a focus on relatively short-term outcomes and reliance on conventional survival analysis, may explain the lack of observed differences. The exclusion of crucial factors such as immunosuppression protocols and long-term considerations could have affected the detection of more nuanced effects on kidney transplant outcomes.

Their findings indicate that CRA could offer more precise estimates of long-term graft survival and death, especially in subgroups of recipients where the impact of competing events is more significant.

This study aims to investigate the impact of kidney transplant center volume on graft loss and patient survival over 3- and 5-year post-transplant periods for adult kidney transplantations in the USA from 2001 to 2015. We implemented CRA guided by a natural biological rationale in this population, selecting a 5-year duration to uncover subtle outcomes over an extended timeframe with increased nuance. Our hypothesis posits that the transplant center's volume significantly influences clinical outcomes, affecting both graft failure and patient mortality. By pre-defining recipients at increased risk for separate subgroup analyses, including diabetic recipients, those receiving high kidney donor profile index (KDPI) deceased donor kidneys, and re-transplant recipients, we aim to unveil the nuanced biological dynamics of these outcomes, with CRA providing enhanced precision, particularly over an extended 5-year follow-up.

4)The authors stated that "We hypothesized that center volume will affect the clinical outcomes including graft failure and patient mortality." I would suggest that the authors provide a rationale for their hypothesis based on the results of the previous studies. Do the authors believe that the negative results of previous studies were due to inappropriate statistical analysis, i.e., the Cox proportional hazards model? As I mentioned above, please consider restructuring the introduction section to better clarify the novelty of the study and the rationale for the hypothesis.

Thank you for your insightful feedback. In response to your suggestion, we have revised our introduction to better elucidate the rationale for our hypothesis. The study now explicitly emphasizes the utilization of Competing Risk Analysis (CRA) based on a natural biological rationale. We have chosen a 5-year follow-up period to capture more nuanced outcomes over an extended timeframe, and we believe this approach enhances the precision of our study. Additionally, we highlight the survival curves in Figure 1, demonstrating the differences even at the 3-year mark, showcasing the effectiveness of CRA in revealing subtle distinctions. We hope these modifications address your concerns and provide a clearer understanding of the study's novelty and rationale.

**Materials and Methods**

5)I would suggest clarifying that the study population was "kidney-only" transplant recipients.

We thank the reviewer for this very insightful suggestion. To clarify, the study indeed focused exclusively on "kidney-only" transplant recipients. To address your comment, we explicitly stated in the revised manuscript that our study was limited to "kidney-only" transplant recipients. This distinction is crucial for the interpretation of our findings and ensures that they are not inadvertently generalized to recipients of other types of organ transplants. We believe that this clarification will enhance the understanding of our study's scope and implications within the specific context of kidney transplantation.

6)I would suggest that "Exposure" and "Outcomes" be described as separate sections.

Thank you for your suggestion. We have revised the manuscript structure as follows:

**Exposure:** Transplant Center Volume: Our primary exposure of interest was the transplant center volume. Transplantation centers were categorized into quartiles (low, medium, medium-high, and high) based on their total volume (divided by years) of kidney-only transplants during the entire study period.

**Outcomes:** 5-Year Graft Failure and Mortality: Our primary outcomes were graft failure and mortality within 5 years post-transplant. Graft failure was defined as a return to dialysis or re-transplantation. Data was censored on December 31, 2020, to allow a five-year follow-up for the study cohort.

**Competing Events Analysis:** Patient Death vs. Graft Failure: Our competing outcomes of interest involved the interplay between patient death and graft failure in the context of kidney transplantation. Graft failure, defined as a return to dialysis or re-transplantation, and patient death were considered as competing measures. Specifically, a patient death was deemed a competing measure if the STAR file contained a record of a date of death alongside a record of a functioning graft. Additionally, it was considered a competing measure when there was no graft failure date or a date indicating the resumption of maintenance dialysis reported. This analysis aims to understand the dynamic relationship between these two critical outcomes in the study cohort.

**Statistical analysis**

7)I would suggest that the authors reconsider the statistical analysis, especially the subgroup analysis of kidney transplant recipients with diabetes. I understood that recipients of living and deceased donor organs were analyzed separately because of the different predicted graft longevity and because of the different covariate adjustment. This statistical approach was conducted in the previous study [1]. However, for the analysis of kidney transplant recipients with diabetes, both living and deceased donors seemed to be included in this study (Table S5). If the authors do not distinguish such recipients, then the recipients of the entire cohort should be included in the main analysis. [1] Sonnenberg EM, Cohen JB, Hsu JY, Potluri VS, Levine MH, Abt PL, et al. Association of kidney transplant center volume with 3-year clinical outcomes. Am J Kidney Dis. 2019; 74(4):441–451.

Thank you for your important feedback regarding the statistical analysis of kidney transplant recipients with diabetes. We recognize the importance of your point about the distinct characteristics and outcomes associated with living and deceased donor organ transplants. We have revised our analysis approach. We now include separate subgroup analyses for diabetic kidney transplant recipients based on the type of donor – those who received organs from living donors and those from deceased donors. The results of these separate analyses are presented in the supplementary materials (Table S3 and S4). This segregation allows for a more nuanced understanding of the competing risks outcomes in these two distinct groups. The findings from these separate analyses have been detailed in the revised manuscript, providing clearer insights into the impact of donor type on diabetic kidney transplant recipients. This modification in our analysis strengthens the study and addresses the concerns you raised, offering a more comprehensive understanding of our study population.

**Table S3.** Competing risk regression models for graft failure and patient death controlling all other covariates (Diabetic patients of living donors).

|  | Graft failure (frailty p-val < 0.001) | | Patient death (frailty p-val < 0.001) | |
| --- | --- | --- | --- | --- |
|  | HR (95% CI) | P-value | HR (95% CI) | P-value |
| Volume groups (ref = “Low”) |  |  |  |  |
| Medium | 1.087 (0.847, 1.395) | 0.514 | 1.001 (0.830, 1.209) | 0.989 |
| Medium-High | 0.880 (0.677, 1.145) | 0.342 | 0.824 (0.677, 1.003) | 0.053 |
| High | 0.838 (0.637, 1.103) | 0.207 | 0.887 (0.735, 1.070) | 0.209 |
| Recipient Age, Years | 0.975 (0.968, 0.983) | <0.001 | 1.040 (1.033, 1.048) | <0.001 |
| Recipient Gender, M | 0.824 (0.693, 0.980) | 0.029 | 0.976 (0.840, 1.135) | 0.755 |
| Recipient Ethnicity (ref = “Caucasian”) |  |  |  |  |
| AA | 1.485 (1.041, 2.118) | 0.029 | 0.917 (0.649, 1.296) | 0.624 |
| Others | 1.140 (0.833, 1.558) | 0.413 | 0.597 (0.444, 0.803) | 0.001 |
| Recipient BMI | 1.019 (1.005, 1.035) | 0.010 | 0.999 (0.986, 1.013) | 0.936 |
| Recipient Peak PRA | 1.004 (1.000, 1.007) | 0.043 | 1.002 (0.999, 1.006) | 0.152 |
| Recipient Kidney CIT | 1.002 (0.983, 1.023) | 0.808 | 1.011 (0.998, 1.024) | 0.105 |
| Recipient Hepatitis C Antibody, Positive | 1.858 (1.293, 2.670) | 0.001 | 1.034 (0.701, 1.523) | 0.867 |
| Recipient Dialysis at TX, Y | 1.317 (1.070, 1.620) | 0.009 | 1.299 (1.102, 1.530) | 0.002 |
| Recipient LOS, Days | 1.010 (1.002, 1.018) | 0.016 | 1.013 (1.008, 1.018) | <0.001 |
| Treated for rejection within 1 year (kidney) | 2.859 (2.337, 3.498) | <0.001 | 1.033 (0.806, 1.323) | 0.799 |
| Donor Age, Years | 1.020 (1.012, 1.028) | <0.001 | 1.004 (0.998, 1.010) | 0.239 |
| Donor Gender, M | 0.979 (0.773, 1.240) | 0.863 | 1.126 (0.927, 1.368) | 0.233 |
| Donor Ethnicity (ref = “Caucasian”) |  |  |  |  |
| AA | 1.015 (0.696, 1.480) | 0.939 | 0.899 (0.622, 1.301) | 0.574 |
| Others | 0.915 (0.656, 1.276) | 0.599 | 0.924 (0.680, 1.256) | 0.614 |
| Donor BMI | 1.008 (0.990, 1.027) | 0.376 | 1.020 (1.004, 1.036) | 0.013 |
| Donor Height | 0.986 (0.975, 0.998) | 0.021 | 0.994 (0.984, 1.003) | 0.207 |
| Donor Hypertension | 1.079 (0.690, 1.689) | 0.738 | 0.935 (0.632, 1.385) | 0.738 |
| HLA antigen mismatch | 1.073 (1.018, 1.130) | 0.009 | 1.029 (0.985, 1.075) | 0.204 |
| Re-transplant | 0.921 (0.660, 1.287) | 0.631 | 1.033 (0.775, 1.378) | 0.825 |
| Transplant year (ref: 2001 – 2005) |  |  |  |  |
| 2006 – 2010 | 0.812 (0.652, 1.01) | 0.062 | 0.874 (0.718, 1.065) | 0.182 |
| 2011 – 2015 | 0.629 (0.501, 0.79) | <0.001 | 0.871 (0.718, 1.058) | 0.164 |
| Immunosuppression |  |  |  |  |
| ATG | 0.958 (0.760, 1.209) | 0.719 | 1.064 (0.872, 1.297) | 0.542 |
| IL-2RA | 0.831 (0.648, 1.067) | 0.146 | 1.086 (0.884, 1.334) | 0.430 |
| CNI | 0.726 (0.552, 0.956) | 0.022 | 0.778 (0.615, 0.983) | 0.036 |
| Steroids | 0.992 (0.812, 1.212) | 0.936 | 1.139 (0.973, 1.334) | 0.105 |

Abbreviations: AA, African American; KDRI, kidney donor risk index; BMI, Body mass index; CIT, Cold ischemia time; LOS, Length of stay; TX: Transplant, IL2-RA: IL2 Receptor Agonist; PRA: Panel Reactive Antibody, Y: Yes

|  | Graft failure (frailty p-val < 0.001) | | Patient death (frailty p-val < 0.001) | |
| --- | --- | --- | --- | --- |
|  | HR (95% CI) | P-value | HR (95% CI) | P-value |
| Volume groups (ref = “Low”) |  |  |  |  |
| Medium | 0.976 (0.87, 1.096) | 0.686 | 0.967 (0.869, 1.076) | 0.537 |
| Medium-High | 0.867 (0.766, 0.981) | 0.024 | 0.858 (0.763, 0.965) | 0.011 |
| High | 0.897 (0.774, 1.038) | 0.144 | 0.957 (0.833, 1.099) | 0.531 |
| Recipient Age, Years | 0.983 (0.979, 0.987) | <0.001 | 1.043 (1.039, 1.046) | <0.001 |
| Recipient Gender, M | 0.925 (0.855, 1.002) | 0.055 | 1.079 (1.005, 1.158) | 0.036 |
| Recipient Ethnicity (ref = “Caucasian”) |  |  |  |  |
| AA | 1.308 (1.197, 1.43) | <0.001 | 0.692 (0.639, 0.750) | <0.001 |
| Others | 0.903 (0.811, 1.006) | 0.064 | 0.611 (0.558, 0.668) | <0.001 |
| Recipient BMI | 1.013 (1.006, 1.02) | <0.001 | 0.991 (0.984, 0.997) | 0.004 |
| Recipient Peak PRA | 1.002 (1, 1.003) | 0.011 | 1.000 (0.999, 1.002) | 0.6 |
| Recipient Kidney CIT | 1.007 (1.002, 1.011) | 0.003 | 1.003 (0.999, 1.007) | 0.213 |
| Recipient Hepatitis C Antibody, Positive | 1.203 (1.011, 1.43) | 0.037 | 1.280 (1.097, 1.493) | 0.002 |
| Recipient Dialysis at TX, Y | 1.237 (1.06, 1.444) | 0.007 | 1.525 (1.337, 1.740) | <0.001 |
| Recipient LOS, Days | 1.005 (1.003, 1.007) | <0.001 | 1.005 (1.004, 1.007) | <0.001 |
| Treated for rejection within 1 year (kidney) | 2.615 (2.386, 2.865) | <0.001 | 1.327 (1.196, 1.472) | <0.001 |
| Donor Age, Years | 1.02 (1.017, 1.023) | <0.001 | 1.003 (1.001, 1.006) | 0.014 |
| Donor Gender, M | 1.06 (0.972, 1.155) | 0.187 | 1.086 (1.008, 1.170) | 0.031 |
| Donor Ethnicity (ref = “Caucasian”) |  |  |  |  |
| AA | 1.472 (1.331, 1.627) | <0.001 | 1.153 (1.046, 1.272) | 0.004 |
| Others | 0.935 (0.835, 1.048) | 0.248 | 0.989 (0.898, 1.090) | 0.824 |
| Donor BMI | 0.993 (0.987, 0.999) | 0.015 | 0.997 (0.992, 1.002) | 0.223 |
| Donor Height | 0.99 (0.988, 0.993) | <0.001 | 0.997 (0.995, 1.000) | 0.039 |
| Donor Diabetes | 1.414 (1.255, 1.593) | <0.001 | 1.136 (1.012, 1.276) | 0.031 |
| Donor Hypertension | 1.261 (1.155, 1.376) | <0.001 | 1.120 (1.035, 1.211) | 0.005 |
| Donor Hep C Antibody, Positive | 1.275 (1.013, 1.604) | 0.038 | 1.213 (0.985, 1.492) | 0.069 |
| Donor Cause of Death (ref = “Other”) | 0.949 (0.75, 1.201) | 0.663 | 1.127 (0.908, 1.399) | 0.276 |
| Anoxia | 0.89 (0.706, 1.121) | 0.322 | 1.102 (0.890, 1.366) | 0.372 |
| Cerebrovascular/Stroke | 0.869 (0.688, 1.096) | 0.236 | 1.030 (0.831, 1.275) | 0.789 |
| Head Trauma | 0.745 (0.421, 1.317) | 0.311 | 1.067 (0.663, 1.719) | 0.788 |
| CNS Tumor | 1.012 (0.899, 1.14) | 0.839 | 1.056 (0.956, 1.166) | 0.285 |
| Donor Don. After Cardiac Death | 1.031 (0.992, 1.072) | 0.118 | 1.028 (0.993, 1.063) | 0.115 |
| Donor Terminal Creatinine | 1.07 (1.044, 1.097) | <0.001 | 1.018 (0.998, 1.038) | 0.083 |
| HLA antigen mismatch | 1.065 (0.917, 1.236) | 0.412 | 1.068 (0.930, 1.225) | 0.351 |
| Re-transplant |  |  |  |  |
| Transplant year (ref: 2001 – 2005) |  |  |  |  |
| 2006 – 2010 | 0.823 (0.745, 0.91) | <0.001 | 0.82 (0.751, 0.896) | <0.001 |
| 2011 – 2015 | 0.678 (0.611, 0.753) | <0.001 | 0.726 (0.662, 0.795) | <0.001 |
| Immunosuppression |  |  |  |  |
| T-cell Depletion Induction | 1.073 (0.964, 1.195) | 0.198 | 1.017 (0.923, 1.12) | 0.735 |
| IL-2RA Induction | 1.09 (0.964, 1.233) | 0.170 | 1.073 (0.965, 1.193) | 0.194 |
| Calcineurin Inhibitor | 0.788 (0.689, 0.901) | 0.001 | 0.85 (0.753, 0.96) | 0.009 |
| Maintenance Steroids | 0.977 (0.89, 1.073) | 0.632 | 1.121 (1.031, 1.22) | 0.008 |

**Table S4.** Competing risk regression models for graft failure and patient death controlling all other covariates (Diabetic patients of deceased donors).

Abbreviations: AA, African American; KDRI, kidney donor risk index; BMI, Body mass index; CIT, Cold ischemia time; LOS, Length of stay; TX: Transplant, IL2-RA: IL2 Receptor Agonist; PRA: Panel Reactive Antibody, Y: Yes

8)I would suggest clarifying the handling of missing data. Given the results of the previous study using Organ Procurement and Transplantation Network registry data [1], I assumed that there were missing data in this study as well. Did you perform a complete case analysis? I would suggest performing the same analysis after multiple imputation of all missing covariates as a sensitivity analysis.

[1] Sonnenberg EM, Cohen JB, Hsu JY, Potluri VS, Levine MH, Abt PL, et al. Association of kidney transplant center volume with 3-year clinical outcomes. Am J Kidney Dis. 2019; 74(4):441–451.

Thank you for your comment regarding the handling of missing data in our study. We initially conducted a complete case analysis. This decision was based on the relatively low level of missingness in our dataset, with most variables having less than 5% missing data. Given the large sample size (n), we deemed this approach to be appropriate and statistically sound for our initial analysis.

However, acknowledging your suggestion and the importance of robustness checks in research, we have revised our manuscript to include additional information regarding missing data. Specifically, we have updated our descriptive tables to explicitly indicate the proportions of missing observations for each covariate. This transparency allows readers to better understand the extent of missingness and its potential impact on our study's findings.

While we did not perform multiple imputations for missing covariates due to the low level of missingness and the large sample size, we agree that it is important to consider such analyses in future studies, especially when missing data is more substantial. This approach would strengthen the robustness of the findings and provide a comprehensive understanding of the impact of missing data on the study outcomes.

We appreciate your suggestion and believe that these revisions will enhance the clarity and rigor of our study's methods and results.

9)Please consider restructuring the statistical analysis section to better clarify its methodology. I understood that the authors conducted competing risk analyses because the authors stated in the introduction section that "Their results show that CRA may provide more accurate estimates of long-term graft survival and death, particularly in subgroups of recipients with higher rates of competing events". In addition, the authors specified "competing risk analysis" in their title. On the other hand, the authors also stated in the statistical analysis section that "We fitted our cause-specific hazards model using the “coxph” function in the “survival” package in R." I would propose to estimate both the cause-specific hazard ratios and the subdistribution hazard ratios.

Thank you for your thoughtful feedback on the statistical analysis section of our manuscript. We understand your point about the need for clarity in the methodology, particularly in relation to the use of competing risk analyses.

In our study, we employed the cause-specific hazards model using the “coxph” function from the “survival” package in R. This is the traditional procedure for conducting cause-specific hazard modeling. This choice was driven by the etiological focus of our study, aiming to understand the factors influencing outcomes in kidney transplant recipients.

However, we appreciate your suggestion to include sub distribution hazard ratios as per the Fine & Gray (1999) model, which is also commonly used in competing risk scenarios. Unfortunately, we encountered significant computational challenges in implementing this approach, using the “cmprsk” package in R, due to the large size of our dataset and its clustered nature (transplants across different centers). The algorithm was found to grind to a halt after running for a few hours on a standard desktop computer. As such, a key limitation of our paper is our inability to present the results of the sub distribution hazards model with a random effect term for the clusters, as would be ideal in this context. This limitation stems from the current state of statistical software and is an area where future advancements are needed. While we recognize the importance of such analyses, the necessary developments in R packages to handle large and complex datasets like ours are beyond the scope of our current research paper. This limitation and the rationale behind our methodological choices are clearly articulated in the revised manuscript. This will provide readers with a complete understanding of the scope and constraints of our statistical analysis.

10) Given that patients who received kidney transplantation between January 2001 and December 2015 were included in this study, an adjustment by year of transplant may be necessary. In addition to immunosuppressive drugs, the quality of transplant team care and surgical techniques has evolved over time.

Thank you for pointing out the importance of considering the year of transplant in our analysis. In response to your suggestion, we have incorporated an adjustment for the year of transplant into our statistical analysis. To effectively capture the changes over time, we have grouped the transplant years into distinct categories: 1-5 years, 5-10 years, and 10-15 years, with the 1–5-year group serving as the reference category. This grouping strategy allows for a nuanced analysis that accounts for the potential impact of medical advancements and policy changes over different periods.

These adjustments and their corresponding impact are now thoroughly detailed in Tables 1-4 and Supplementary Tables 2, 3, 4, S1, and S2 of our revised manuscript. By including these year groupings as covariates, we ensure a more accurate and comprehensive analysis that reflects the temporal dynamics in kidney transplantation practices and outcomes.

We believe this addresses your insightful comment regarding the time-related variables in kidney transplant outcomes.

**Results**

11)When using competing risk analysis, I would suggest that the adjusted "sub distribution hazard ratio" be stated instead of the adjusted "hazard ratio" throughout the manuscript to avoid confusing the readers.

Thank you for your suggestion regarding the terminology in our manuscript. To enhance clarity and avoid any potential confusion among readers, we will ensure that the term "adjusted cause-specific hazard ratio" is consistently used throughout the manuscript when discussing results derived from the competing risk analysis based on the statistical approach. This change will differentiate these findings from those obtained through other methods, such as the Cox proportional hazards model, where the term "adjusted hazard ratio" is appropriate. Your feedback is invaluable in improving the precision and readability of our manuscript.

**Discussion**

12) I would propose to revise the text regarding the summary of results in the first paragraph of discussion section as follows: we report that, compared to low (Q1) volume centers, medium-high (Q3) volume (median: 121 transplants/year) US transplant centers had better outcomes. The Q1 center volume was set as the reference throughout the analysis.
Thank you for this valuable suggestion. We have revised the text in the first paragraph of the discussion section as follows:

In this large retrospective observational study using a novel statistical competing risks design, we find that transplant center volume has an independent effect on both long-term graft loss as well as patient death. We report that, compared to low (Q1) volume centers, medium-high (Q3) volume (median: 121 transplants/year) US transplant centers had better outcomes. The Q1 center volume was set as the reference throughout the analysis.

13)I would suggest that the authors further discuss the differences in outcomes between living and deceased donor kidney transplantation. Why was kidney transplant center volume not associated with both graft loss and mortality in living donor kidney transplant recipients? Could this discrepancy be due to differences in the graft survival of living and deceased donor kidney transplant recipients?

Thank you for guiding us in enhancing the discussion section.

The lack of association between kidney transplant center volume and both graft loss and mortality in LDKT recipients may be attributed to several factors. One plausible explanation could be that LDKT procedures are often characterized by different dynamics and considerations compared to deceased donor procedures. Living donor kidney transplants typically involve healthier organs and donors, leading to generally favorable outcomes. Additionally, LDKTs are often planned and scheduled in advance, allowing for meticulous donor and recipient matching, and optimized perioperative care. The distinct nature of LDKT, with factors such as pre-screening of donors and controlled timing, may diminish the impact of center volume on outcomes. In contrast, DDKT involves a different set of challenges and considerations.

**Figure**

14)In the Kaplan-Meier curve, I would suggest that the number of recipients in each group be listed at the bottom of the curve.

Certainly, listing the number of recipients at the bottom of the Kaplan-Meier curve is a valuable suggestion to enhance clarity and transparency in the presentation of the data. This addition will provide readers with a clear understanding of the sample size for each group and contribute to the overall interpretation of the Kaplan-Meier analysis. Thank you for this insightful suggestion; in this revision, we have incorporated it into the presentation of the Kaplan-Meier curve in the manuscript.

**Table**

**15)**I would suggest that the number of recipients in each group be listed at the top of the table. For example, Low (Q1) (n = ????).

We thank the reviewer for this thoughtful suggestion. The manuscript has been revised to reflect the number of recipients in each quartile group at the top of the relevant tables.

16)As I mentioned above, I would suggest that the results of the competing analysis and the Cox proportional hazards model be described side by side. I am interested in whether the results of the two differ from each other.

We acknowledge the reviewer’s suggestion to compare the results of the sub-distribution hazards model with the cause-specific hazards model under competing risk analysis. However, due to the limitations of existing statistical software in handling sub distribution hazard models with random effects for large, clustered datasets, such a comparison was not feasible in our study. We have outlined these computational challenges in our revised manuscript to provide clarity on the scope and limitations of our analysis.

**Reviewer #3 Comments:**

1.This research topic is interesting and worth discussing. There are significant differences in baseline variables that can affect outcome depending on the center volume. Although many variables have been statistically adjusted in the current analysis, since the center volume is a variable in which different variables are combined, it cannot be completely free from the difference between the values of the baseline variable. The statistical analysis using propensity score matching seems appropriate to fully confirm the impact of the center volume. It would be more informative to present the results of the main outcome analyzed by propensity score matching as supplementary data.

Thank you for your insightful suggestion and your valuable time in reviewing our study.

In observational studies, propensity score matching is mostly used to compare multiple groups or treatments, considering target endpoints such as usual right-censored events or competing risks. In our analysis, the emphasis is not on comparing specific groups or treatments; instead, we focus on the overall risk evaluation of available risk factors on the target time to event (i.e., time to graft failure), which is subject to competing risks. Hence, we opted against using the propensity score matching approach.

2. How many missing data was there for each variable and how was it handled during statistical analysis?

Thank you for your inquiry regarding the handling of missing data in our study. Initially, we conducted a complete case analysis, considering the relatively low level of missingness in our dataset, where most variables had less than 5% missing data. Given the substantial sample size (n), we deemed this approach appropriate and statistically sound for our initial analysis.

However, in response to your valuable suggestion and recognizing the importance of robustness checks, we have updated our manuscript. Descriptive tables now explicitly indicate the proportions of missing observations for each covariate, offering transparency about the extent of missingness and its potential impact on our study's findings.

Although we did not perform multiple imputations for missing covariates due to the low level of missingness and the large sample size, we acknowledge the importance of considering such analyses in future studies, particularly when missing data is more substantial. This approach would enhance the robustness of findings and provide a more comprehensive understanding of the impact of missing data on study outcomes.

We appreciate your insightful recommendation and believe these revisions will improve the clarity and rigor of our study's methods and results.

**Reviewer #4 Comments:**

Dear Editor and authors, thank you for the opportunity to review this interesting manuscript.

This is a 5-year retrospective cohort analyzed by competing risks for graft failure and death affected by center volume. Please, see below my comments:

Major considerations

Introduction

Kindly review previously published prediction models for similar outcomes in graft loss or death kidney transplant recipients by center volume capacity or other administrative aspects.

Thank you for your thorough review and valuable comments on our manuscript. We have carefully considered your suggestions and made revisions to address the major considerations you raised.

Introduction:

A major focus of research in the field of kidney transplantation is to address factors that can compromise the overall goal of kidney transplantation. Transplant center volume is one such factor that has been a topic of debate in transplant community.^1,2^ Previous studies have shown that high volume kidney transplant centers had better short-term outcomes at 1 month and 1 year post-transplant, presumably because of better peri-operative surgical technique and management experience.^1-3^ Transitions of care from transplant center to local nephrology are variable from center-to-center and may be impacted by center volume with higher volume centers transitioning patients earlier due to manpower limitations. The impact on outcomes of specialized longitudinal medical care and the availability of a collaborative healthcare team, that is more likely to be available at transplant center level rather than with community nephrologist; has not been well studied.^4^ While it has been shown that an increasing number of dialysis patients per nephrologist can have an adverse impact on outcomes, no such data exists for kidney transplant patients being taken care of at transplant centers.^5^ These problems are further complicated by a dearth of both the nephrology and the transplant nephrology workforce in the United States.^6,7^

Sonnenberg et al categorized kidney transplant centers into low, medium, medium-high, and high volume based on the mean annual number of kidney transplants from 2009 to 2013. Their findings indicated no evidence of improved outcomes with increased center volume^8^. The study's limitations, including a focus on relatively short-term outcomes and reliance on conventional survival analysis, may explain the lack of observed differences. The exclusion of crucial factors such as immunosuppression protocols and long-term considerations could have affected the detection of more nuanced effects on kidney transplant outcomes.

Recent publications suggest that conventional survival analysis methods ignoring the competing event(s), such as Kaplan–Meier (KM) method and standard Cox proportional hazards regression, may be inappropriate in the presence of competing risks, and alternative methods specifically designed for analyzing competing risks data should be considered.^9,10^ This problem deserves more attention in prognostic research in patients with advanced kidney disease, mainly because an important assumption of KM is that the censoring event (death or graft loss) is an independent event with independent probability value.^9^ It means that subjects that are censored at a certain time point are expected to be as likely to experience the endpoint of interest as those that are not censored.^9^ An alternative method used to estimate survival probabilities is the competing risk analysis (CRA) which requires no assumption about independence between the two risks, graft loss and death. In addition, Ters et al have shown that factors like aging and diabetes that are generally associated with higher mortality should be considered as competing events for death in kidney transplant patients while analyzing long-term outcomes.^11^ Their findings indicate that CRA could offer more precise estimates of long-term graft survival and death, especially in subgroups of recipients where the impact of competing events is more significant.

This study aims to investigate the impact of kidney transplant center volume on graft loss and patient survival over 3- and 5-year post-transplant periods for adult kidney transplantations in the USA from 2001 to 2015. We implemented CRA guided by a natural biological rationale in this population, selecting a 5-year duration to uncover subtle outcomes over an extended timeframe with increased nuance. Our hypothesis posits that the transplant center's volume significantly influences clinical outcomes, affecting both graft failure and patient mortality. By pre-defining recipients at increased risk for separate subgroup analyses, including diabetic recipients, those receiving high kidney donor profile index (KDPI) deceased donor kidneys, and re-transplant recipients, we aim to unveil the nuanced biological dynamics of these outcomes, with CRA providing enhanced precision, particularly over an extended 5-year follow-up.

Materials and Methods

Study population:

¿Were patients with primary renal graft thrombosis (arterial or venous) excluded?

No patients, irrespective of whether they experienced primary renal graft thrombosis in the arterial or venous vessels, were excluded from the study. Your inquiry for clarification is appreciated.

Statistical analysis:

The methods section should clarify the modeling technique and etiology/predictor variable definition.

Please mention the performance model of this CRA analysis

It is advisable consultation of the STROBE and TRIPOD statements on reporting of observational studies and prediction models, respectively.

Does it have this model internal or external validation? please include the technique to validate or justify the lack of internal/external validation?

Thank you for your insightful comments. We have thoroughly revised the statistical analysis section in compliance with the STROBE guidelines, ensuring clarity and transparency. While we did not conduct internal or external validation for the CRA model, we have included a reference showcasing the application of similar models in the kidney disease and transplant population.

Your guidance has been immensely helpful, and we appreciate the opportunity to enhance the robustness of our study.

Results

Is the increase of the survival rates in patient death with graft failure significant in the diabetic patient's group compared to patient death not from kidney graft failure? Are there differences between the other groups?

Please include the results of the internal validation

Please include the results of the performance model

Discussion

Please amplify the results of Sonnenberg et al. for a better understanding of this comparison.

Please explain the utility of this final etiology model and how it could be incorporated into some other aspects of the health system or clinical use.

Thank you for your feedback regarding the statistical analysis of kidney transplant recipients with diabetes. We appreciate the importance of understanding the distinct characteristics and outcomes associated with living and deceased donor organ transplants.

In response to your suggestion, we have revised our analysis approach. Separate subgroup analyses for diabetic kidney transplant recipients based on the type of donor—living and deceased—have been incorporated into our study. The detailed results of these separate analyses are now presented in the supplementary materials (Table S3 and S4). This segregation allows for a more nuanced understanding of the competing risks outcomes in these two distinct groups.

Regarding the internal validation, we want to clarify that we did not perform internal validation for our study. While internal validation could provide additional insights, the decision not to conduct it was based on the specific context of our research, including the large sample size and the nature of the data.

We appreciate your thorough review and believe that these modifications enhance the comprehensiveness and robustness of our study.

Many thanks for your valuable input and consideration!

Minor considerations

Discussion

The word “arenas” should be replaced by areas. Please verify. In the sentence “A similar association is seen in almost all surgical arenas where it could be said that ‘practice indeed makes perfect”

Thank you for pointing out the term "arenas" in the Discussion section. We have reviewed the sentence, and considering the context, we believe the term "arenas" is appropriate. In this context, "arenas" refers to various fields or domains where surgical practices occur. It suggests that the observed association is consistent across different surgical contexts or areas.

However, we understand the concern, and if you prefer, we can replace "arenas" with "areas" to ensure clarity. Please let us know your preference.

A heartfelt thank you to all the reviewers for their invaluable time and insightful feedback. Your contributions have been instrumental in enhancing the quality of our work. We sincerely appreciate your thorough review and constructive comments.
